# Lubrication behavior of ex-vivo salivary pellicle influenced by tannins, gallic acid and mannoproteins

**DOI:** 10.1016/j.heliyon.2022.e12347

**Published:** 2022-12-17

**Authors:** Georgios Agorastos, Olaf van Nielen, Emo van Halsema, Elke Scholten, Aalt Bast, Peter Klosse

**Affiliations:** a Faculty of Science and Engineering Department, Maastricht University, Nassaustraat 36, 5911 BV, Venlo, the Netherlands; b T.A.S.T.E. Foundation, Garstkampsestraat 11, Overasselt, 6611 KS, the Netherlands; c Physics and Physical Chemistry of Food, Wageningen University, Bronse Weilanden 9, Wageningen, the Netherlands

**Keywords:** Tribology, Saliva, Mouthfeel, Astringency, Aggregate, Diameter

## Abstract

The objective of this study was to investigate the influence of tannins and gallic acid on the salivary lubrication behavior. Furthermore, the effects of pH and mannoproteins in combination with gallic acid on the lubrication of saliva were studied. The addition of gallic acid and tannins were found to increase friction caused by the removal of the saliva film. Tannins resulted in higher friction compared to gallic acid. Lowering pH increased friction of gallic acid mixtures with saliva, due to stronger interactions between gallic acid and saliva. The increased friction caused by gallic acid was inhibited by the addition of mannoproteins due to the hydrogen bond interactions between gallic acid and mannoproteins, thereby decreasing the complex formation between gallic acid and salivary proteins. A correlation of 0.96 was found between the hydrodynamic diameter of the aggregate and the delta friction suggesting that the formation of aggregates determined the lubrication behavior.

## 1 Introduction

Mouthfeel plays a significant role in the sensory experience of alcoholic or nonalcoholic beverages, like wine, beer, or tea (Gawel et al., 2018). In beverages, mouthfeel contributes to the perceived texture and taste perception. This sensation arises partly from the influence of polyphenols on the integrity of the saliva film that coats oral mucosa (Laguna et al., 2017).

Astringent mouthfeel is an important indicator of the sensory quality of a wide range of beverages (Bajec and Pickering, 2008). Astringency is commonly associated with a drying mouthfeel, even though it is a very complex sensation with various definitions and origins (receptor and lubrication based). Astringency perception in beverages has been associated with plant-based polyphenols (Gawel et al., 2018). These polyphenols interact with salivary proteins through non-covalent interactions and result in the depletion of the lubricating salivary film (Rudge et al., 2021). Loss of salivary lubrication has been reported to be related to the astringency sensation (Bajec and Pickering, 2008). When mucins and proline-rich proteins have been suggested as the most important salivary proteins for oral lubrication (Rudge et al., 2021).

Tannins and gallic acid are some of the main phenolic components that interact with salivary proteins. Gallic acid is known to contribute to the astringency of beverages like red wines, while tannins are perceived as more astringent components compared to gallic acid (Frank et al., 2011; Hufnagel and Hofmann, 2008). Since tannins contain larger molecules than gallic acids such as catechins, ellagitannins etc, the molecular weight influences the astringency perception (Ma et al., 2016). Additionally, phenols concentration and the wine matrix (like ethanol, pH, and polysaccharides) are important parameters for the impact on the astringency perception (Gawel et al., 2018).

The mechanisms behind astringent perception are not completely known and are still under debate. At the moment, two mechanisms have been suggested to explain astringency sensation, the receptor and friction-based mechanism (Canon et al., 2021). The most discussed one is the friction-based mechanism, by which astringency is explained by the interaction of tannins with specific salivary proteins (Charlton et al., 2002). This theory suggests that mucins, proline-rich proteins (PRPs) and histatins readily interact with tannins (Bennick, 2002). The mucus layer in the mouth consists of salivary proteins that are non covalently bound to oral mucosal cells. Green (1993) suggested that the astringent sensation is mainly caused by the interaction of polyphenols with salivary proteins forming insoluble polyphenols-proteins precipitates (via cross-linking) in the mouth. Those aggregates increase the friction in the oral cavity and reduce the lubrication of the salivary film, giving an astringent/dry sensation. The interaction of polyphenols and salivary proteins is suggested to happen in three stages (Jöbstl et al., 2004). The first stage is the interaction between the aromatic rings of polyphenols and the hydrophobic sites (pyrrolidine ring of the proline residues) of the salivary proteins to form soluble complexes. In the second stage, the protein-polyphenols complexes self-associate via hydrogen bonding to form larger soluble complex aggregates. The peptide groups (carboxyl and -NH_2_) of the salivary proteins are cross-linked with the addition of extra polyphenol hydroxyl groups via hydrogen bonding. In the last stage, these complex aggregates grow further until they become insoluble, and are large enough to precipitate and induce phase separation.

The formation of the above-mentioned aggregates between polyphenols and salivary proteins depends on a variety of parameters, such as the ratio between proteins and polyphenols, pH, temperature, ionic strength, and the type of polyphenol (Bajec and Pickering, 2008). Even though the formation of aggregates, which leads to loss of lubrication, is considered the main precursor to astringency sensations, researchers have suggested that astringency can also arise without the occurrence of such interactions. This supports that astringency is a more complicated sensation that depends on more than one single physical or chemical mechanism (Rossetti et al., 2009). Therefore, a receptor-based theory suggests the astringency sensation can be origin from direct interaction with the salivary proteins adhered to buccal mucosal cells (Canon et al., 2021).

The beverage matrix is a rich composition of many elements. Depending on the composition, the matrix can induce or reduce astringent perception. For instance, polysaccharides or proteins in wine (like mannoproteins and arabinogalactan) may influence the astringency sensation as they have been shown to disrupt the interaction and aggregation between salivary proteins and polyphenols (Watrelot et al., 2017). The pH is another astringency modulator that may affect the interactions. Previous studies have shown that the astringency sensation increases (Gawel et al., 2018). This increase in astringency sensation may be related to an increase in phenol groups, which may form hydrogen bonds with salivary proteins (Rudge et al., 2021).

An emerging tool that helps understanding oral processing is tribology (Stokes et al., 2013). Tribology is the science of wear, friction and lubrication and includes how interacting surfaces and other tribo-elements behave in relative motion. Soft-tribology can mimic aspects of in-mouth lubrication by applying human saliva and soft surfaces. The use of soft-tribology helps to study mouthfeel characteristics (such as astringency) by simply monitoring the friction change. Previous studies related to the mouthfeel properties of tea, milk, and wine have used tribology to scale the astringency sensations (Laguna et al., 2017). Even though lubrication behaviour is important for the understanding of astringency perception, limited studies have focused on the mechanisms behind lubrication losses.

The present work aims to investigate the effect of gallic acid and tannins on the lubrication properties of human saliva at different pH levels. Furthermore, the masking effect of mannoproteins in the lubrication properties was tested. The outcome of this study provides new insights into the effect of different conditions on the lubrication properties of human saliva. The outcome of this research has the potential to help the food industry in influencing the beverage matrix and the astringent sensation.

## 2 Material and methods

### 2.1 Model solutions

The model solutions (MS) were made based on astringent components that can be found in wine. Samples were prepared with or without the presence of gallic acid (Sigma-Aldrich Corp, St. Louis, MO, USA), tannins (65% w/w total polyphenols in gallic acid equivalent, 38% w/w total catechin equivalent Lamothe-Abiet, France), a 15% w/v mannoprotein solution produced by grape yeast (Lamothe-Abiet, Bordeaux, France), potassium dihydrogen phosphate, and meta-phosphoric acid (Merck Millipore, Darmstadt, Germany). Demineralized water was used as the solvent for all MS. All samples were formulated on the same day as the analysis in duplicate. The concentrations of gallic acid range between 0.5 and 4 g/L. The mannoproteins had a constant concentration of 400 mg/L for all the gallic acid combinations. All samples were covered with aluminum foil and stored at 4 °C for a maximum of 24 h to prevent degradation. Gallic acid and tannin concentrations were chosen to represent both the presence of compounds as well as the values of the total amount of polyphenols that can be found in wines (Büyüktuncel et al., 2014; Petković et al., 2015).

To investigate the effect of pH, the components of the MS were diluted in demineralized water or a phosphate buffer at pH 3. A phosphate buffer was used instead of tartaric acid since it has been recognized as an astringent compound (Huang and Xu, 2021). The pH 3 was selected since represents the most acidic beverages. The buffer solution was prepared by complete dissolution of 0.34% (w/v) potassium hydrogen phosphate in demineralized water. A phosphoric acid solution (1M) was used to adjust the pH to 3.

### 2.2 Saliva collection

It is suggested that there are no fluids capable of mimicking the properties of real human saliva (Stokes and Davies, 2007). Therefore, for both tribological and aggregate formation measurements, fresh unstimulated human saliva was provided by healthy non-smoking donors, four male subjects (age 22–25, Caucasian) after their consent. This physiological group was selected to minimize the variation in the composition of saliva (Xu et al., 2019). This part of the study has been approved by the Ethical Review Committee at Maastricht University [ethics reference (ERCIC_335_23_03_2022)].

The saliva collection followed by a procedure described by Rudge and co-workers (Rudge et al., 2021). To minimize the variation of the human saliva due to circadian rhythms through the day (Dawes, 1975), the collection took place between 8 and 10 am. Consumption of food was avoided for at least 2 h before collection. The saliva was collected without stimulation of the salivary glands (Brossard et al., 2016). During salivating, the first milliliters of saliva were discarded as possible contaminants could still be present in the mouth. The saliva was collected and stored on ice to prevent degradation by enzymes. After collection, saliva was centrifuged at 10000 rpm (9520 g) at 4 °C for 10 min to remove remaining debris. The supernatant was stored in ice and used immediately after centrifugation since the viscoelasticity of saliva decreases during storage (Stokes and Davies, 2007).

### 2.3 Tribological measurements

A dynamic tribological approach was used to measure the changes in the frictional coefficient of saliva upon the addition of the MS. All tribological measurements were performed with an Anton Paar Rheometer MCR302 (Austria). A tribology cell (BC12.7/SS 52837) was used to measure the lubrication properties of the samples in combination with saliva. Polydimethylsiloxane (PDMS) pins were used since PDMS is a prevailing material currently used in tribology (Rudge et al., 2021). The friction was measured using a commercial (glass) ball on a three PDMS pin setup. The glass ball had a diameter of 12.7 mm and PDMS pins a diameter of 6 mm and a height of 6 mm with a modulus of around 2 MPa. Glass ball and PDMS pins were obtained by the rheometer manufacturer.

The measurements were performed in triplicates. A normal force, Fn, of 1 N was applied (Laguna et al., 2017). The experiments were carried out at a constant rotational speed of 1 mm/s to gain boundary regime friction profiles, as this regime is believed to be closely related to the perception of astringency in humans (Prakash et al., 2013). Similarly to the protocol used by Rudge et al. (2021) the measurements were taken within a period of 10 min, where the first 5 min were used for the salivary proteins to cover the PDMS pins (ex-vivo salivary pellicle). The salivary layer allows the glass probe to slide against the PDMS pins while lubricating by 0.5 mL of saliva. When the 5 min passed and a constant friction coefficient was obtained, the MS were added in a 1:1 (saliva: MS) ratio as has been suggested by other researchers (Laguna et al., 2017; Rudge et al., 2021).

**Figure 1:**
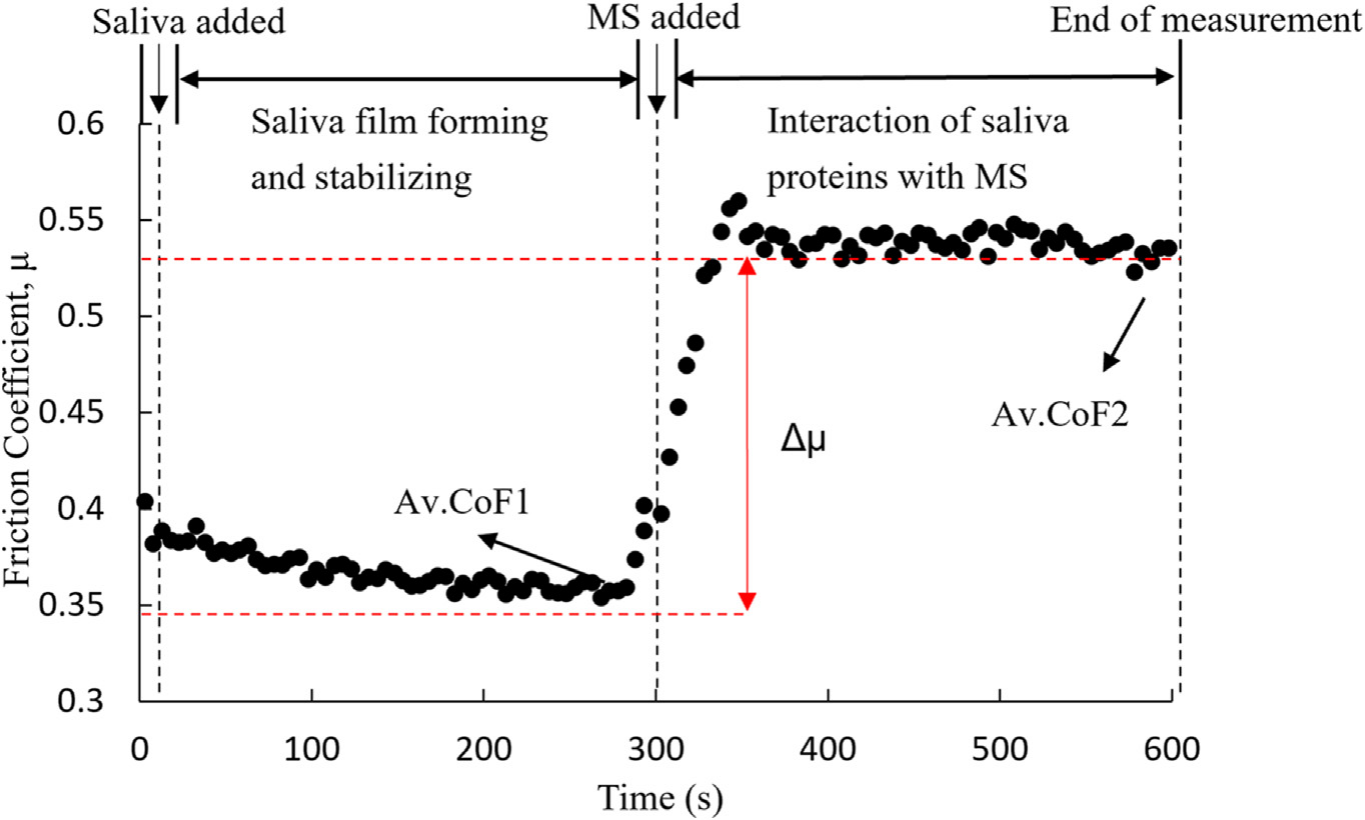
A schematic representation of the friction coefficient, μ, as a function of time obtained by the dynamic protocol. Saliva was added at time 0 of the measurement. After obtaining a constant baseline value as the result of saliva addition (AvCoF_1_), the model solution (MS) was added after 300 s. The Av.CoF_2_ was calculated using only the data points after stabilization of the interaction between saliva and MS stabilized.

An example of measurement is shown in Figure 1. With the use of these graphs, the difference in friction coefficient (Δμ) was calculated as Av.CoF_1_–Av.CoF_2,_ where Av.CoF_1_ is the friction coefficient obtained when salivary proteins fully covered the PDMS surface, and Av.CoF_2_ is the friction coefficient after the addition of the MS. All the friction coefficients represent mean values by taking each average value based on five points.

### 2.4 Zeta-potential and particle size distribution

Zeta-potential and particle size distribution were measured by dynamic light scattering using a Zetasizer Ultra (Malvern Instruments). The particle size distribution was measured in triplicate (173° Backscatter), with a refractive index (RI) of 1.450 for the protein, and an RI of 1.330 for the water. First, pure saliva and astringent compounds solutions were measured. Afterwards, Saliva-MS of 0.9 ml (1:1) were mixed in Eppendorf tubes for 5 min at 20 °C. The samples were diluted 100 times prior to measurement. For the zeta potential determination, the samples were tested 3 times at a maximum voltage of 60 mV for a maximum of 100 runs per test. The cuvettes were cleaned between each measurement with demi-water, ethanol, and again demi-water and subsequently dried with pressurized air. The measuring cell was changed every 5 samples because of electrolysis (blackening) of the electrodes.

### 2.5 Statistical analysis

The results were analyzed using a multivariate analysis of variance (MANOVA) and a Pearson correlation. When the values from MANOVA were significantly different (p < 0.05), an additional Tukey-Kramer HSD (honestly significant difference) test was used to identify the differences between the parameters. To verify the assumptions of normal distribution and homogeneity of variances, Shapiro-Wilk test and Levene's test were used. All the statistical analyses were performed by the software R (R core team and foundation for statistical computing), R-studio version 4.0.3 using the R package “agricolae”.

## 3 Results and discussion

### 3.1 Differentiation of the influence of tannins and gallic acid on lubrication properties of human saliva

**Figure 2:**
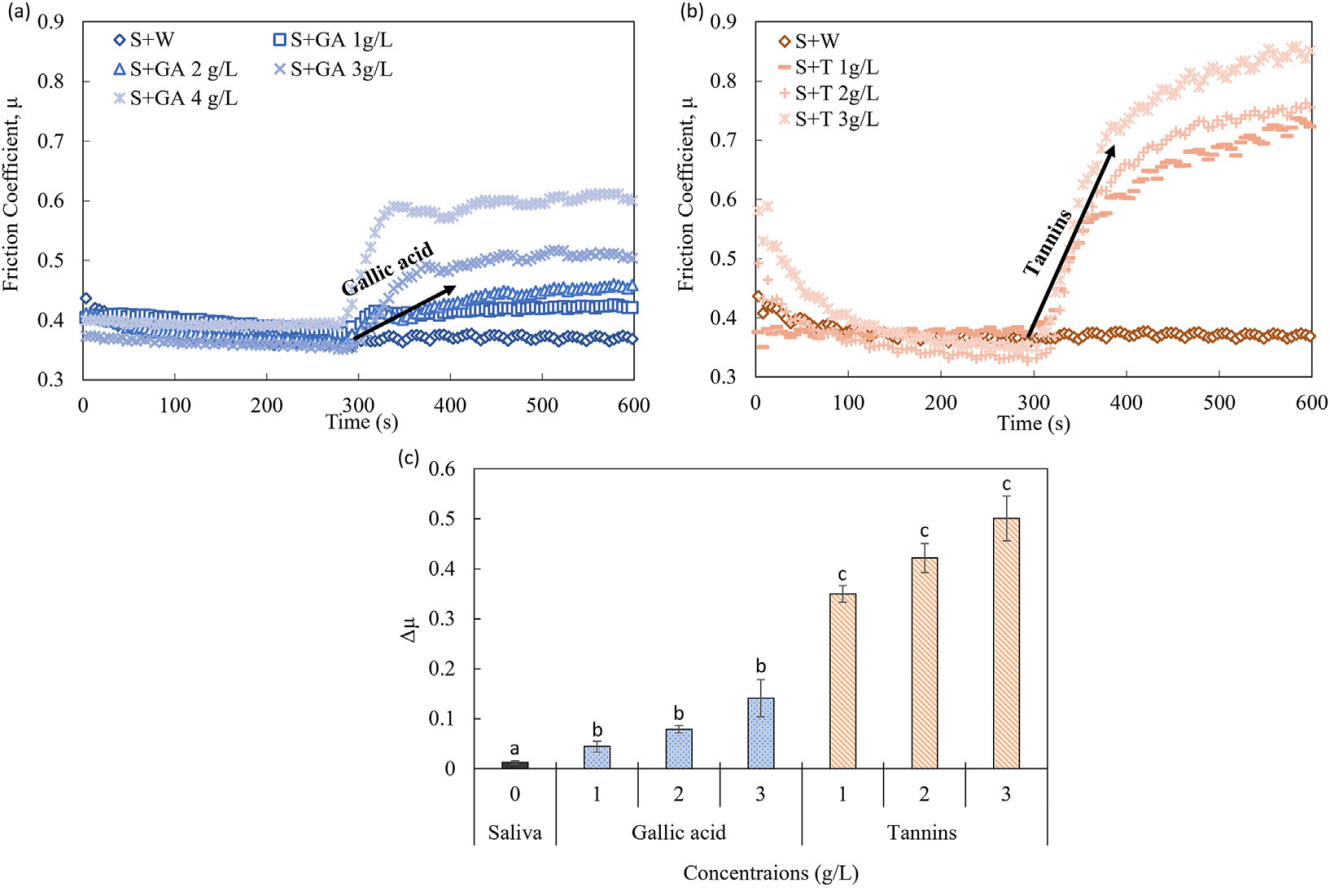
Friction coefficient as a function of time for gallic acid (GA) (a) and tannins (T) (b) in demi-water solution at different concentrations mixed with human saliva in a 1:1 ratio. W refers to water, and S refers to saliva. The friction coefficients represent mean values based on five measurement points. (c) Δμ values as a function of saliva, gallic acid and tannins concentrations. Gallic acid (dotted bars) and tannins (striped bars) solutions were mixed with human saliva in a 1:1 ratio. The values are presented as a mean value ±standard error. The letters ^a-c^ in each bar identify that the samples are significantly different according to Tukey's HSD test: p < 0.05.

Changes in the lubrication behavior of saliva are associated with interactions between salivary proteins and polyphenols. Gallic acid and tannins are components that are abundantly present in beverages like wine, tea, etc, and are known to provide an astringent sensation. Those components are different in molecular weight, which is expected to influence lubrication. The change in the saliva lubrication behavior upon the addition of gallic acid and tannins was investigated in this study. Different concentrations of gallic acid and tannins were used to identify patterns in the increase of friction. The results are shown in Figure 2a and b.

Within 5 min, the friction coefficient reached a constant value (baseline) due to the formation of a saliva film on the substrate. The friction coefficient was found to give a constant value of on average 0.37. Such lubrication properties have been found previously, with saliva friction coefficients ranging between 0.25 and 0.35 at low loading forces (Chen, 2009).

The low friction coefficient for saliva is associated with the adsorption of salivary proteins on the PDMS surface. The hydrophobic nature of the PDMS provides good adhesion for the salivary proteins (Carpenter et al., 2019). Upon the addition of the MS, i.e. gallic acid and tannin solutions, the saliva film loses its lubrication properties and friction increases (Figure 2a and b). As can be seen, tannins lead to a larger increase in the friction coefficient than gallic acid. The molecular weight of the astringent compounds seems to influence the lubrication properties of saliva. Additionally, for both components, a clear effect of concentration was also observed.

To gain better insights into the effect on the friction coefficients upon the addition of the different astringent agents, the Δμ values after the addition of the MS were determined. These results are given in Figure 2c. To examine any water potential effect upon the addition of the MS, demi-water was used as a reference. The addition of demi-water into saliva gave a Δμ value around 0, indicating that water itself did not change the salivary lubrication.

Noticeable is the difference between the two astringent compounds gallic acid and tannins (Figure 2c). Upon addition of both gallic acid and tannins, Δμ significant increased (p < 0.001) with gallic acid and tannins. However, gallic acid solutions gave low values for Δμ of 0.044, 0.078 and 0.141 for concentrations of 1, 2 and 3 g/L, respectively, while the Δμ values for tannins were significantly higher at values of 0.349, 0.421 and 0.501 for concentrations of 1, 2 and 3 g/L, respectively. Changes in friction of saliva upon addition of polyphenols were demonstrated by other researchers as well (Watrelot et al., 2017). Additional observation between the tannin and gallic acid solutions was obtained regarding the different rates of the changes in lubrication behavior. Similar to friction changes, the rates were higher in tannin's presence compared to gallic acid.

The differences in the Δμ values can be explained by the difference in the polyphenol-protein interactions. Those interactions are mainly driven by two types of interactions, i.e., hydrogen bonds and hydrophobic interactions. Hydrophobic stacking interactions occur via the binding sites of the peptides (proline residues) together with the preceding amide bond and amino acid, between the galloyl ring (phenolic compounds) and the pyrrolidine ring face of proline (García-Estévez et al., 2018). Proline residues are the main binding sites of the salivary proteins for the hydrophobic interactions with phenolic compounds. Additionally, the hydrogen bonds form between the hydroxyl groups of the phenolic compounds and the carbonyl and amino groups of the salivary protein groups. The last-mentioned interactions are believed to stabilize the formation of the aggregates between salivary proteins and phenolic compounds (Charlton et al., 2002).

As these interactions are more pronounced for compounds with a larger molecular weight, the difference in Δμ between the gallic acid and tannin solutions can be explained by the molecular weight of the components. As the tannins are larger, they are expected to form more bonds with multiple salivary proteins, i.e. PRPs, mucins, statherin and P–B peptide, which leads to the formation of larger aggregates. Such relation was also suggested by Laguna et al. (2017), who showed that the aggregate size, obtained by interactions between oak tannins and salivary proteins, increased astringency perception. Therefore, we believe the increase in friction observed in our study can be related to an increase in astringency perception upon consumption.

The current findings show that molecular weight affects the absolute change in the friction coefficient, as a result of differences in the interactions between the astringent components and the salivary proteins.

### 3.2 Effect of pH on the lubrication properties of human saliva in combination with a gallic acid series

Not only the molecular weight of polyphenols is important for astringency perception in beverages, but pH is another parameter that has been found to influence astringency. To investigate the influence of pH on the lubrication behavior, gallic acid was selected, as this astringent compound was found to give more reproducible results than tannins, since the lower solubility of tannins in buffer solution and sample inhomogeneity (data not shown).

**Figure 3:**
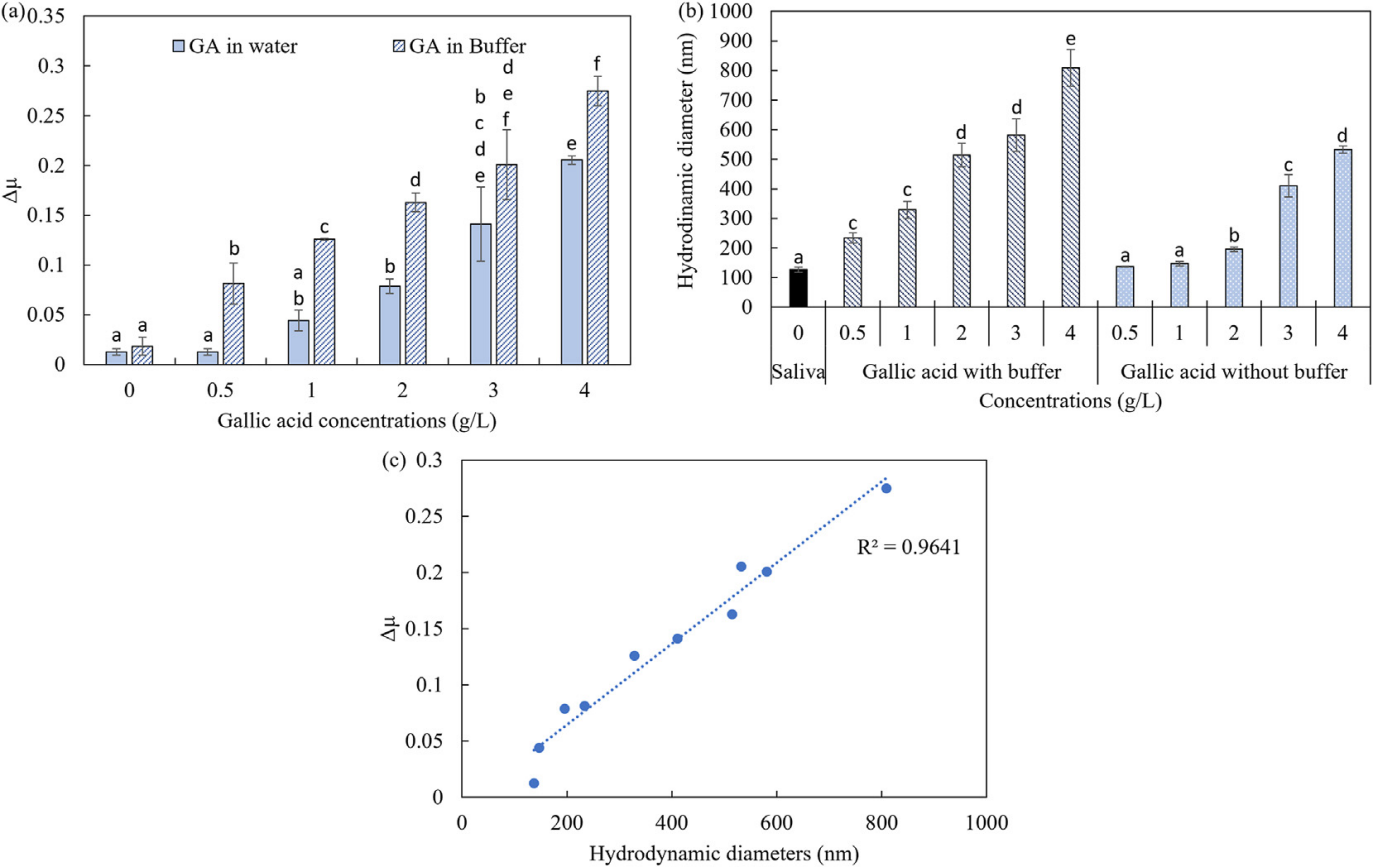
(a) Δμ values as a function of gallic acid concentration for gallic acid (GA) diluted in water (solid bars) and phosphate buffer (pH 3) (striped bars). All the solutions were added in the presence of ex vivo salivary film. (b) The hydrodynamic diameter of saliva–gallic acid aggregates in buffer (striped bar) and without buffer (dotted bars). The values are presented as a mean value ±standard error. The letters a–e in each bar identify that the samples are significantly different according to Tukey's HSD test: p < 0.05. (c) Δμ as a function of hydrodynamic diameter for gallic acid. The line is the best through the data points.

The gallic acid solutions were diluted either in demi-water or a buffer solution of pH 3. A significant increase in friction (p < 0.001) upon the addition of gallic acid to ex vivo saliva pellicle was noticed for both gallic acid solutions prepared with demi-water and phosphate buffer solutions (Figure 3a). In both solutions, the gallic acid caused an increase in the friction coefficient, presented as Δμ, in the presence of ex vivo salivary film, as already discussed in the previous section.

The addition of buffer solution alone in ex vivo saliva pellicle was not able to significantly increase the delta friction coefficient, as can be seen in Figure 3a. However, in the presence of gallic acid, a clear effect of pH on changes in the friction coefficient can be observed. Addition of gallic acid diluted in a pH 3 buffer gave significant higher Δμ values (p < 0.001) than addition of gallic acid diluted in demi-water when added to saliva. Especially for the lowest concentration of GA, the effect of pH was clear. The low concentrations of 0.5 and 1 g/L showed a significant difference between the two solutions. This shows that the effect of pH is especially important for low concentrations of polyphenols. This may be related to differences in the aggregate formation of salivary proteins at these different pH values.

To verify that indeed the aggregate formation was influenced by changes in pH, we measured the size of the aggregates, as the hydrodynamic diameter, formed by salivary proteins and gallic acid solutions (Figure 3b). As can be observed, the hydrodynamic diameter of human salivary proteins was around 127 nm. This value is similar to the findings presented by Laguna et al. (2017). The addition of gallic acid into saliva (1:1) resulted in a significant (p < 0.001) increase in the hydrodynamic diameter for all the concentrations in both buffer and demi-water solutions. This increase in size confirms the aggregate formation between the salivary proteins and gallic acid, for which the more pronounced aggregate formation was observed in the buffer solution. This indeed confirms that more aggregation was obtained at lower pH values.

In water, a significant (p < 0.001) increase in the aggregate formation was obtained only at or above a concentration of 2 g/L. In buffer solutions, the increase was significant for all concentrations, and the increase in the hydrodynamic diameter seems to be linear with gallic acid concentrations. At the highest gallic acid concentration of 4 g/L the largest diameter of 809 nm was observed. The aggregate formation of both series was found to correlate with the Δμ values, as shown in Figure 3c. The Pearson's correlation between the Δμ and aggregate size for gallic acid was 0.96 (p < 0.05) respectively. These results show that higher friction can indeed be associated with aggregate formation. As fewer salivary proteins attached to the PDMS surface are available, no salivary proteins are available to provide lubrication in the oral cavity.

The higher Δμ and larger aggregate size for lower pH values (in buffer) can be explained by the different interactions. Changes in pH will affect the binding affinity by altering the charge and configuration of the protein. Repulsive electrostatic interaction may limit the attractive hydrophobic interactions and hydrogen bond formation. Salivary proteins at physiological pH carry a negative electrical charge (Rykke et al., 1995). Mucins are responsible for this net negatively charge since their isoelectric point is around 2.5 (Veerman et al., 1992). At lower pH values, the electrostatic repulsion between mucins and polyphenol reduces as the charge density of the mucins reduces at pH values close to the iso-electric point. Once the repulsive interaction decreases, the attractive interactions become more relevant, which induces aggregate formation between proteins and polyphenols.

To investigate the effect of pH on the charge of salivary proteins and gallic acid, we measured the zeta-potential and pH of the resulting protein-gallic acid complexes/aggregates. The pure saliva had a pH of 7.3 and a zeta potential of −15.5 mV. Upon the addition of the buffer solution of pH 3, the pH decreased to a value of 5.8 and the zeta-potential was −15.6 mV (Table 1). The phosphate buffer at pH 3 failed to bring the pH to a value of 3 due to the high buffering ability of saliva (Bardow et al., 2000). The addition of gallic acid decreased the pH values in the buffer solution. The pH values of the mixtures (saliva-MS) decreased from 5.8 to 3.9 when the gallic acid concentration was increased to 4 g/L. Because of the pH reduction, the zeta-potential of the mixtures changed from −15.6 to −13.9 mV. In water, the gallic acid solutions did not decrease the pH for low concentrations, but only at high gallic acid concentrations (3 and 4 g/L), the pH change was substantial: the pH decreased from 7.3 without gallic acid to 4.7 with 4 g/L gallic acid. Also, the zeta-potential was only slightly lower for higher concentrations of gallic acid. Based on these differences in the zeta-potential between the MS in water and buffer we conclude that the salivary proteins become less charged at lower pH values. Less negatively charged proteins could potentially aggregate easier due to less electrostatic repulsion between gallic acid and proteins. The lower net electrical charge in the buffer solutions confirms the more aggregate formation between salivary proteins and polyphenols. This contributes to the higher loss of salivary lubrication behavior.

**Table 1:** pH and zeta-potential values of pure saliva or mixtures of saliva with model solutions with and without buffer solutions.

pH 3 buffer	Mixture	pH	Zeta-potential (mV)
Without buffer	**Saliva**	7.3 ± 0.2	−15.5 ± 0.1
	**Saliva GA (0.5 g/L)**	6.8 ± 0.1	−15.8 ± 0.6
	**Saliva GA (1 g/L)**	6.4 ± 0.1	−15.5 ± 0.1
	**Saliva GA (2 g/L)**	6.0 ± 0.1	−14.6 ± 0.1
	**Saliva GA (3 g/L)**	4.9 ± 0.1	−13.9 ± 0.7
	**Saliva GA (4 g/L)**	4.7 ± 0.1	−13.8± 1.2
Buffer	**Saliva**	5.8 ± 0.1	−15.6 ± 0.1
	**Saliva GA (0.5 g/L)**	5.4 ± 0.1	−13.9 ± 0.9
	**Saliva GA (1 g/L)**	4.6 ± 0.1	−14.0 ± 1.4
	**Saliva GA (2 g/L)**	4.4 ± 0.1	−13.9 ± 0.9
	**Saliva GA (3 g/L)**	4.1 ± 0.1	−13.6 ± 0.3
	**Saliva GA (4 g/L)**	3.9 ± 0.1	−13.9 ± 0.3

Remark: The values are presented in mean value ±standard deviation.

Although the overall charge of the complexes/aggregates decreased, the charge of the different proteins in the saliva is not the same, due to differences in their structure. Mucins contribute up to 30% of the proteins present in saliva, while PRPs contribute the other 70% (Carpenter et al., 2019). PRPs can be categorized into basic, acidic (bPRPs), acidic (aPRPs)and glycosylated (gPRPs), of which the basic and glycosylated have the highest proline content. The isoelectric point of mucins is around 2.5 (Veerman et al., 1992), of acidic PRPs around 4.5 (McArthur et al., 1995), of bPRPs around 9.5, and that of gPRPs around 7 (Boze et al., 2010). At physiological pH (around 7), mucins and acidic PRPS are thus responsible for the negative charge of saliva. However, at lower pH values, the acidic PRPs become more neutral and the charge of mucins also decreases (Veerman et al., 1992). In addition, gallic acid has a pKa value of around 4.4. At lower pH values, gallic acid therefore also becomes less charged, which was also confirmed by the lower zeta-potential of −2.1 mV when present in the buffer solution.

As both gallic acid and mucins became less charged at lower pH, the electrostatic repulsion was minimized, and attractive hydrogen bonds and hydrophobic interactions became more pronounced. These interactions increased aggregate formation and resulted in higher Δμ values in most acidic conditions. In contrast, for higher pH values, a higher charge provided more electrostatic repulsion and thus less aggregate formation, resulting in a lower change in the friction coefficient.

Other studies have also shown the influence of pH on saliva protein-polyphenol interactions (Rinaldi et al., 2012). Those studies focused mostly on the impact of pH on aggregate formation and astringency perception. However, few studies tested the effect of pH on the lubrication properties of saliva (Vardhanabhuti et al., 2011; Wang et al., 2020). In a study by Vardhanabhuti et al. (2011), the effect of β-lactoglobulin on the aggregation behavior at two different pH values (3.5 and 7) was studied. They observed that the changes in pH influence the interaction between β-lactoglobulin and salivary proteins; at pH 3.5, more aggregate formation was observed and higher friction. In the presence of tannins, Wang et al. (2020) showed that a faster collapse of the salivary pellicle was obtained at lower pH. However, Wang et al. (2020) did not find a significant difference in the resulting friction coefficients between different pH values. Those friction-based studies demonstrate the further need for a better understanding of the mechanisms behind the changes in salivary lubrication.

Our current results provide new insights on the salivary lubrication loss upon the addition of gallic acid influenced by pH. Lower pH in combination with gallic acid leads to more aggregate formation. A linear relationship was found between concentration and Δμ values in low pH. Those changes in friction due to pH are in line with already mentioned studies related to higher astringency perception and aggregate formation due to lower pH. Lastly, we can conclude that the extent of lubrication loss is not only due to the size of the polyphenols but also to parameters that directly influence the aggregate formation, such as pH.

### 3.3 Masking effect of mannoproteins

Astringent components are known to alter the lubrication of saliva by aggregating salivary proteins. However, components like mannoproteins can provide a “masking” effect by inhibiting the aggregation, and thus reduce astringent perception (Wang et al., 2021). Mannoproteins are known to disrupt the interaction between salivary proteins and polyphenols (Watrelot et al., 2017). However, it is not known how mannoproteins influence the lubrication behaviour of saliva. As aggregate formation and lubrication losses were more pronounced in a buffer solution of pH 3, we investigated whether mannoproteins would be able to reduce this effect under these conditions.

Mannoproteins are proteins located in the outermost layer of the yeast cell wall. These proteins are naturally present in wine and known to mask astringency. Mannoproteins are glycoproteins with a polysaccharide backbone representing about 80% of the molecule, which is highly abundant in mannose monomer residues (Gonçalves et al., 2002). About 20% of the molecule consists of protein residues, which are linked to the polysaccharide part via amide bonds at asparagine amino acid residues, or ether bonds at serine or threonine residues (Moreno and Peinado, 2012).

**Figure 4:**
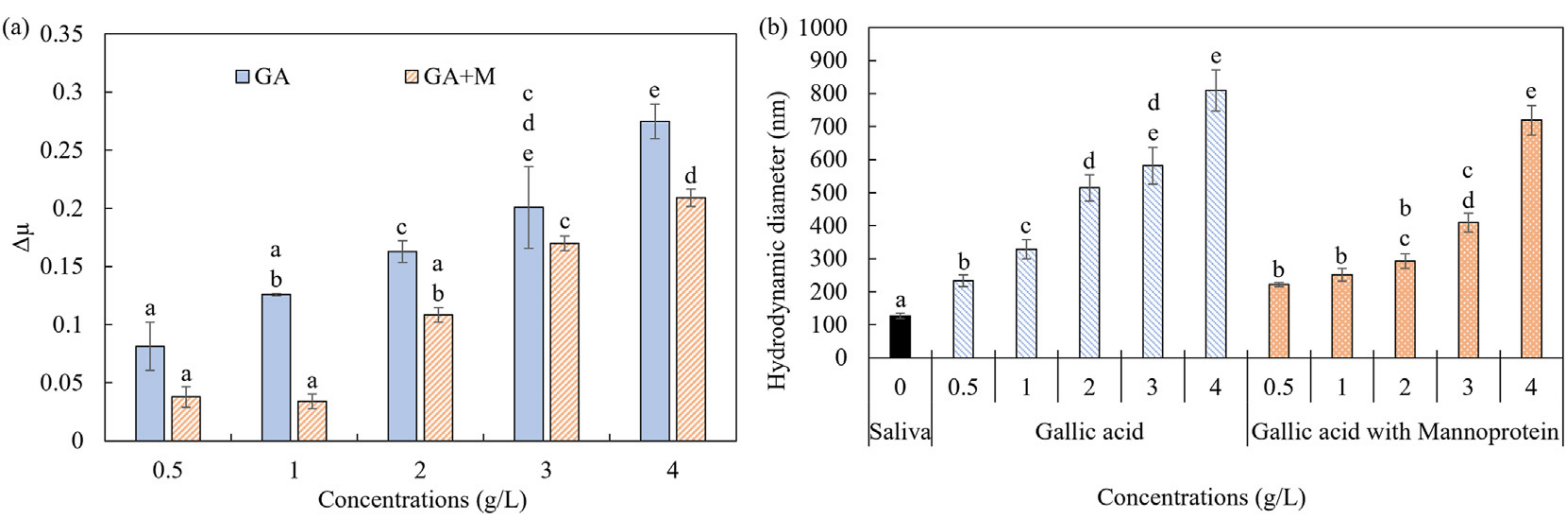
(a) Δμ values as function of gallic acid concentration for gallic acid (GA) (solid bars) and gallic acid plus mannoproteins (M) (striped bars). All the solutions were added in the presence of an ex vivo salivary film. (b) Hydrodynamic diameter of pure saliva (solid bar), saliva–gallic acid combinations (striped bars) against saliva–gallic acid–mannoproteins (dotted bars). All the solutions were diluted in pH 3. The values are presented as a mean value ±standard error. The letters a–e in each bar identify that the samples are significantly different according to Tukey's HSD test: p < 0.05.

The addition of mannoproteins solution (400 mg/L) itself resulted in a higher friction coefficient (0.45) compared to saliva (0.37). The effect of the addition of mannoproteins to gallic acid systems regarding the Δμ values is shown in Figure 4a. The mannoproteins had a constant concentration of 400 mg/L for all the gallic acid combinations. This concentration was chosen to represent the concentration of mannoproteins in wine (Wang et al., 2021). The addition of mannoproteins in the gallic acid solutions gave a significant decrease in the delta friction values (p < 0.001). It seems that at lower concentrations of gallic acid of 0.5 and 1.0 g/L, the mannoprotein was able to provide stronger masking of friction, i.e. lower values of Δμ, than at higher concentrations of gallic acid. This indicates that there is a specific amount of binding affinity of mannoproteins to gallic acid.

These results indeed indicate the ability of mannoproteins as inhibitors for lubrication loss. However, two main mechanisms have been suggested to explain the reduction of astringency by mannoproteins and polysaccharides. The first mechanism suggests the formation of protein/polyphenol/mannoprotein ternary soluble complexes (Manjón et al., 2020; Ramos-Pineda et al., 2018). The other mechanism states that only interactions between polyphenols and mannoproteins occur (Brandão et al., 2017). To investigate the exact mechanism that occurs in our study, we measured the hydrodynamic diameter (aggregate size) and the zeta-potential of the MS with and without mannoproteins.

Figure 4b presents the hydrodynamic diameter (nm) for pure saliva, different concentrations of gallic acid and the addition of mannoprotein in gallic acid solutions with different concentrations. The diameter showed a similar trend with Δμ (Figure 4a), for which the addition of gallic acid increased the diameter, while with the addition of mannoproteins, the diameter was smaller.

The addition of mannoprotein resulted in a significant (p < 0.001) decrease in the hydrodynamic diameter. For lower gallic acid concentrations of 0.5 and 1 g/L, this effect was minimal, but the decrease of the aggregated salivary proteins became more noticeable at 2 g/L. At this concentration, the aggregates of gallic acid-salivary proteins had a diameter of 515 nm without mannoprotein, but this size decreased to 292 nm with the addition of mannoprotein. The decrease of the hydrodynamic diameter was again smaller for a concentration of 4 g/L, as the original size of 809 nm decreased to 719 nm only. Although again a relation could be observed between Δμ and the hydrodynamic diameter, the correlation was less pronounced (0.873).

It was observed that at low gallic acid concentrations, the addition of mannoproteins did not significantly increase the diameter of the aggregates. Contrary, Δμ was significantly reduced at low gallic acid concentrations by the addition of mannoproteins. Additionally, at higher gallic acid concentrations, both the aggregate diameter and Δμ values were slightly smaller upon the addition of mannoproteins. Therefore, the prevention of salivary lubrication loss, induced by mannoproteins, can be explained partly by the aggregate size but also by the interactions between gallic acid and mannoproteins. Those results suggest that smaller aggregate sizes induce less friction in the system. It seems that in our case the phenol and the mannoprotein interact with each other before aggregating salivary proteins through hydrogen bond interactions. This can explain the similar aggregate size but lower friction at low gallic acid concentrations with and without the addition of mannoproteins. The current outcome suggests that the second-mentioned masking mechanism (interaction between phenol and mannoproteins) applies in our case.

The masking ability of mannoproteins can be further explained by the affinity of mannoproteins to bind with phenolic components, with or without salivary proteins. Since gallic acid and mannoproteins interact with each other via hydrogen bond, there are less “free” gallic acid molecules that can interact with the salivary proteins. Therefore, when the gallic acid concentration exceeds the limit of the binding ability of mannoproteins, the remaining “free” gallic acid forms aggregates with the salivary proteins, which leads to the decrease of the salivary lubrication properties. For a better understanding of the binding between mannoproteins, gallic acid and/or salivary proteins, the zeta-potential and pH values of the samples were measured.

The addition of mannoprotein in gallic acid-saliva solutions gave a slight increase in the pH values of the solutions compared to gallic acid-saliva solutions without mannoproteins (Table 2). The pH values for solutions with mannoproteins varied between 6.1 and 4.0 for concentrations of gallic acid of 0.5 and 4 g/L respectively, whereas pH values without mannoproteins varied between 5.4 to 3.9. Additionally, the zeta-potential values were slightly affected by the addition of mannoprotein (Table 2). The zeta-potential values in the mannoproteins solutions were lower compared to the values without mannoproteins. This difference can be explained by the negative charge of the mannoproteins since mannoproteins in buffer pH 3 had a zeta-potential of −4.1 mV. This shows that the presence of mannoproteins lowers the zeta potential of the gallic acid solutions but the effect was minimal.

**Table 2:** Zeta-potential and pH values after mixing with human saliva (1:1) with different concentrations of gallic acid (buffer pH 3) with and without mannoproteins (1:1).

Mannoproteins (g/L)	Gallic acid (g/L)	pH	Zeta-potential (mV)
0	**0.5**	5.4 ± 0.1	−13.9 ± 0.9
	**1**	4.6 ± 0.1	−14.1 ± 1.4
	**2**	4.4 ± 0.1	−13.9 ± 0.9
	**3**	4.1 ± 0.1	−13.6 ± 0.3
	**4**	3.9 ± 0.1	−13.9 ± 0.3
0.4	**0.5**	6.1 ± 0.1	−15.7 ± 0.1
	**1**	5.4 ± 0.2	−15.1 ± 0.6
	**2**	4.6 ± 0.1	−13.8 ± 0.1
	**3**	4.4 ± 0.1	−15.1 ± 1.5
	**4**	4.0 ± 0.1	−14.4 ± 0.3

Remark: The values are presented in mean value ±standard deviation.

The small differences in the zeta-potential suggested that the electrostatic interactions are not dominant for the binding inhibition between salivary proteins and gallic acid via mannoprotein. The smaller aggregate formation is therefore not related to electrostatic effects but must be related to other interactions. The main interactions that facilitate the lower binding between gallic acid and salivary proteins are more related to hydrogen bond formation or hydrophobic interactions. We expect that the mannoproteins can bind to the gallic acid molecules through both types of interactions, which then prevents the gallic acid molecules to bind with the salivary proteins. This would thus explain the decrease in the hydrodynamic radius of the aggregates.

The hydrogen bonds form between the hydroxyl-groups of the gallic acid with the oxygen from the sugar linkages of the mannose, which account for 80% of the mannoproteins (Casassa, 2017). Hydrophobic interactions may occur as well, but are expected to be less prominent. As the mannoproteins consist of only 20% of proteins, of which only approximately 9% of the amino acid residues of yeast mannoproteins have an aromatic ring (Liu et al., 2015), the binding affinity through hydrophobic interactions is expected to be limited. This indicates the importance of hydrogen bonds for the aggregate formation between gallic acid and (manno)proteins.

Our current results suggest that mannoproteins can inhibit the binding between gallic acid and salivary proteins, as the addition of mannoproteins resulted in less aggregate formation. The formation of ternary protein/gallic acid/mannoprotein complexes is thus less likely to occur. The inhibition of the aggregate formation allows salivary proteins to lubricate the oral surfaces. This results in lower Δμ values, even though large aggregates are still obtained. Especially at low concentrations of gallic acid, where mannoproteins can bind almost completely to all gallic acid molecules. We thus propose that the mechanism for the “masking” is caused by the complex formation of gallic acid and mannoproteins only, which leaves the salivary proteins available for lubrication. This is in contrast with the different studies that suggest that ternary complexes are formed. This shows that the masking effect of mannoproteins may vary with different phenolic components. This can be due to differences in the molecular structure, as this may influence the affinity of mannoproteins to bind with phenolic components, which may determine whether they only aggregate with the astringent components, or also with saliva together.

Our findings give new insights into the masking effect of mannoproteins on gallic acid-induced lubrication loss. We revealed the influence of mannoproteins on the aggregate formation between gallic acid and salivary proteins. The current results are in line with sensory studies, and thus indicate the importance of salivary lubrication behavior in understanding astringency perception. The involvement of other wine components should be further investigated. This would provide a better understanding of the mechanism behind the “masking” effect of friction.

## 4 Conclusion

Lubrication properties of saliva, during food consumption, are known to be correlated with astringency sensation. This study investigated the effect of tannins and gallic acid on the lubrication properties of saliva. Tannins and gallic acid were found to reduce salivary lubrication. Tannins lead to a significantly higher increase in friction than gallic acid. pH was shown to have a significant effect, as the charge of the components determined the degree of aggregation. Less electrostatic repulsion between salivary proteins and polyphenols at lower pH values increased the degree of aggregation, which was shown to be linearly related to changes in friction.

Mannoproteins provided a masking effect for the lubrication loss. Mannoproteins showed affinity to bind with astringent components to create gallic acid-mannoprotein complexes. The addition of mannoproteins, therefore, inhibited the aggregation of salivary proteins with gallic acid, thereby providing enough salivary lubrication ability. The changes in salivary lubrication losses could further link to astringency perception.

This study gives insights into the interactions between salivary proteins and polyphenols that occur during food and beverage consumption. The current outcomes show that instrumental lubrication analysis can be a valuable tool for investigating mouthfeel sensations. This is important for the design of new products or product reformulation.

## Declarations

### Author contribution statement

Georgios Agorastos: Conceived and designed the experiments, Performed the experiments, Wrote the paper, Analyzed and interpreted the data.

Olaf van Nielen: Performed the experiments, Analyzed and interpreted the data.

Emo van Halsema, Elke Scholten, Aalt Bast, Peter Klosse: Conceived and designed the experiments, Analyzed and interpreted the data, Contributed reagents, materials, analysis tools or data.

### Funding statement

Peter Klosse was supported by Province of Limburg, The Netherlands (SAS-2017-03583).

### Data availability statement

The data that has been used is confidential.

### Declaration of interest’s statement

The authors declare no competing interests.

### Additional information

Supplementary content related to this article has been published online at [URL].

## References

[bib1] Bajec M.R., Pickering G.J. (2008). Astringency: mechanisms and perception. Crit. Rev. Food Sci. Nutr..

[bib2] Bardow A., Moe D., Nyvad B., Nauntofte B. (2000). The buffer capacity and buffer systems of human whole saliva measured without loss of CO2. Arch. Oral Biol..

[bib3] Bennick A. (2002). Interaction of pant polyphenols with salivary proteins. Crit. Rev. Oral Biol. Med..

[bib4] Boze H., Marlin T., Durand D., Pérez J., Vernhet A., Canon F., Sarni-Manchado P., Cheynier V., Cabane B. (2010). Proline-rich salivary proteins have extended conformations. Biophys. J..

[bib5] Brandão E., Silva M.S., García-Estévez I., Williams P., Mateus N., Doco T., de Freitas V., Soares S. (2017). The role of wine polysaccharides on salivary protein-tannin interaction: a molecular approach. Carbohydr. Polym..

[bib6] Brossard N., Cai H., Osorio F., Bordeu E., Chen J. (2016). Oral” tribological study on the astringency sensation of red wines. J. Texture Stud..

[bib7] Büyüktuncel E., Porgalı E., Çolak C. (2014). Comparison of total phenolic content and total antioxidant activity in local red wines determined by spectrophotometric methods. Food Nutr. Sci..

[bib8] Canon F., Belloir C., Bourillot E., Brignot H., Briand L., Feron G., Lesniewska E., Nivet C., Septier C., Schwartz M., Tournier C., Vargiolu R., Wang M., Zahouani H., Neiers F. (2021). Perspectives on astringency sensation: an alternative hypothesis on the molecular origin of astringency. J. Agric. Food Chem..

[bib9] Carpenter G., Bozorgi S., Vladescu S., Forte A.E., Myant C., Potineni R.V., Reddyhoff T., Baier S.K. (2019). A study of saliva lubrication using a compliant oral mimic. Food Hydrocolloids.

[bib10] Casassa L.F. (2017). Phenolic Compounds - Natural Sources, Importance and Applications.

[bib11] Charlton A.J., Baxter N.J., Khan M.L., Moir A.J.G., Haslam E., Davies A.P., Williamson M.P. (2002). Polyphenol/peptide binding and precipitation. J. Agric. Food Chem..

[bib12] Chen J. (2009). Food oral processing—a review. Food Hydrocolloids.

[bib13] Dawes C. (1975). Circadian rhythms in the flow rate and composition of unstimulated and stimulated human submandibular saliva. J. Physiol..

[bib14] Frank S., Wollmann N., Schieberle P., Hofmann T. (2011). Reconstitution of the flavor signature of dornfelder red wine on the basis of the natural concentrations of its key aroma and taste compounds. J. Agric. Food Chem..

[bib15] García-Estévez I., Ramos-Pineda A.M., Escribano-Bailón M.T. (2018). Interactions between wine phenolic compounds and human saliva in astringency perception. Food Funct..

[bib16] Gawel R., Smith P.A., Cicerale S., Keast R. (2018). The mouthfeel of white wine. Crit. Rev. Food Sci. Nutr..

[bib17] Gonçalves F., Heyraud A., de Pinho M.N., Rinaudo M. (2002). Characterization of white wine mannoproteins. J. Agric. Food Chem..

[bib18] Green B.G. (1993). Oral astringency: a tactile component of flavor. Acta Psychol..

[bib19] Huang R., Xu C. (2021). An overview of the perception and mitigation of astringency associated with phenolic compounds. Compr. Rev. Food Sci. Food Saf..

[bib20] Hufnagel J.C., Hofmann T. (2008). Orosensory-directed identification of astringent mouthfeel and bitter-tasting compounds in red wine. J. Agric. Food Chem..

[bib21] Jöbstl E., O’Connell J., Fairclough J.P.A., Williamson M.P. (2004). Molecular model for astringency produced by polyphenol/protein interactions. Biomacromolecules.

[bib22] Laguna L., Sarkar A., Bryant M.G., Beadling A.R., Bartolomé B., Victoria Moreno-Arribas M. (2017). Exploring mouthfeel in model wines: sensory-to-instrumental approaches. Food Res. Int..

[bib23] Liu H.-Z., Liu L., Hui H., Wang Q. (2015). Structural characterization and antineoplastic activity of *Saccharomyces cerevisiae* mannoprotein. Int. J. Food Prop..

[bib24] Ma S., Lee H., Liang Y., Zhou F. (2016). Astringent mouthfeel as a consequence of lubrication failure. Angew. Chem..

[bib25] Manjón E., Brás N.F., García-Estévez I., Escribano-Bailón M.T. (2020). Cell wall mannoproteins from yeast affect salivary protein–flavanol interactions through different molecular mechanisms. J. Agric. Food Chem..

[bib26] McArthur C., Sanson G.D., Beal A.M. (1995). Salivary proline-rich proteins in mammals: roles in oral homeostasis and counteracting dietary tannin. J. Chem. Ecol..

[bib27] Moreno J., Peinado R. (2012).

[bib28] Petković B.B., Stanković D., Milčić M., Sovilj S.P., Manojlović D. (2015). Dinuclear copper(II) octaazamacrocyclic complex in a PVC coated GCE and graphite as a voltammetric sensor for determination of gallic acid and antioxidant capacity of wine samples. Talanta.

[bib29] Prakash S., Tan D.D.Y., Chen J. (2013). Applications of tribology in studying food oral processing and texture perception. Food Res. Int..

[bib30] Ramos-Pineda A.M., García-Estévez I., Dueñas M., Escribano-Bailón M.T. (2018). Effect of the addition of mannoproteins on the interaction between wine flavonols and salivary proteins. Food Chem..

[bib31] Rinaldi A., Gambuti A., Moio L. (2012). Precipitation of salivary proteins after the interaction with wine: the effect of ethanol, pH, fructose, and mannoproteins. J. Food Sci..

[bib32] Rossetti D., Bongaerts J.H.H., Wantling E., Stokes J.R., Williamson A.-M. (2009). Astringency of tea catechins: more than an oral lubrication tactile percept. Food Hydrocolloids.

[bib33] Rudge R.E.D., Fuhrmann P.L., Scheermeijer R., van der Zanden E.M., Dijksman J.A., Scholten E. (2021). A tribological approach to astringency perception and astringency prevention. Food Hydrocolloids.

[bib34] Rykke M., Smistad G., Rölla G., Karlsen J. (1995). Micelle-like structures in human saliva. Colloids Surf. B Biointerfaces.

[bib35] Stokes J.R., Boehm M.W., Baier S.K. (2013). Oral processing, texture and mouthfeel: from rheology to tribology and beyond. Curr. Opin. Colloid Interface Sci..

[bib36] Stokes J.R., Davies G.A. (2007). Viscoelasticity of human whole saliva collected after acid and mechanical stimulation. Biorheology.

[bib37] Vardhanabhuti B., Cox P.W., Norton I.T., Foegeding E.A. (2011). Lubricating properties of human whole saliva as affected by β-lactoglobulin. Food Hydrocolloids.

[bib38] Veerman E.C.I., van den Keybus P.A.M., Valentijn-Benz M., Nieuw Amerongen A.v. (1992). Isolation of different high-Mr mucin species from human whole saliva. Biochem. J..

[bib39] Wang S., Olarte Mantilla S.M., Smith P.A., Stokes J.R., Smyth H.E. (2020). Astringency sub-qualities drying and pucker are driven by tannin and pH – insights from sensory and tribology of a model wine system. Food Hydrocolloids.

[bib40] Wang S., Olarte Mantilla S.M., Smith P.A., Stokes J.R., Smyth H.E. (2021). Tribology and QCM-D approaches provide mechanistic insights into red wine mouthfeel, astringency sub-qualities and the role of saliva. Food Hydrocolloids.

[bib41] Wang S., Wang X., Zhao P., Ma Z., Zhao Q., Cao X., Cheng C., Liu H., Du G. (2021). Mannoproteins interfering wine astringency by modulating the reaction between phenolic fractions and protein in a model wine system. LWT.

[bib42] Watrelot A.A., Schulz D.L., Kennedy J.A. (2017). Wine polysaccharides influence tannin-protein interactions. Food Hydrocolloids.

[bib43] Xu F., Laguna L., Sarkar A. (2019). Aging-related changes in quantity and quality of saliva: where do we stand in our understanding?. J. Texture Stud..

