# Supplementary material for: Lubrication behavior of ex-vivo salivary pellicle influenced by tannins, gallic acid and mannoproteins
Source: Heliyon. 2022 Dec 17;8(12):e12347. doi: 10.1016/j.heliyon.2022.e12347 (PMC9793261; doi:10.1016/j.heliyon.2022.e12347)
Supplement: Supplementary material [file mmc1.docx]

Table S1. Product data sheet of skin tannins from Lamothe-Abiet.

| Product : | Skin tannins |
| --- | --- |
| Issue Date: | 12-11-2019 |
| Appearance: | Granulate brown |
| Phenolic content (as % gallic acid equivalent): | 65 % |
| Humidity: | <10 % |
| Ashes: | <4 % |
| Iron: | <50 mg/kg |
| Arsenic: | <3 mg/kg |
| Mercury: | <1 mg/kg |
| Lead: | <5 mg/kg |
| Insoluble matter (water): | <2 % |
